# Succinate dehydrogenase and MYC-associated factor X mutations in pituitary neuroendocrine tumours

**DOI:** 10.1530/ERC-22-0157

**Published:** 2022-08-08

**Authors:** Paul Benjamin Loughrey, Federico Roncaroli, Estelle Healy, Philip Weir, Madhu Basetti, Ruth T Casey, Steven J Hunter, Márta Korbonits

**Affiliations:** 1 Patrick G Johnston Centre for Cancer Research, Queen’s University, Belfast, UK; 2 Regional Centre for Endocrinology and Diabetes, Royal Victoria Hospital, Belfast Health & Social Care Trust, Belfast, UK; 3 Geoffrey Jefferson Brain Research Centre, Division of Neuroscience and Experimental Psychology, School of Medicine, Manchester University, Manchester, UK; 4 Department of Cellular Pathology, Royal Victoria Hospital, Belfast Health & Social Care Trust, Belfast, UK; 5 Department of Neurosurgery, Royal Victoria Hospital, Belfast Health & Social Care Trust, Belfast, UK; 6 Cancer Research UK Cambridge Institute, Li Ka Shing Centre, University of Cambridge, Cambridge, UK; 7 Department of Endocrinology, Cambridge University Hospital NHS Foundation Trust, Cambridge, UK; 8 Centre for Endocrinology, William Harvey Research Institute, Barts and The London School of Medicine and Dentistry, Queen Mary University of London, London, UK

**Keywords:** succinate dehydrogenase, SDH, MAX, pituitary neuroendocrine tumour, paraganglioma, phaeochromocytoma

## Abstract

Pituitary neuroendocrine tumours (PitNETs) associated with paragangliomas or phaeochromocytomas are rare. *SDHx* variants are estimated to be associated with 0.3–1.8% of PitNETs. Only a few case reports have documented the association with *MAX* variants. Prolactinomas are the most common PitNETs occurring in patients with *SDHx* variants, followed by somatotrophinomas, clinically non-functioning tumours and corticotrophinomas. One pituitary carcinoma has been described.* SDHC, SDHB* and* SDHA* mutations are inherited in an autosomal dominant fashion and tumorigenesis seems to adhere to Knudson’s two-hit hypothesis. *SDHD* and *SDHAF2* mutations most commonly have paternal inheritance. Immunohistochemistry for SDHB or MAX and loss of heterozygosity analysis can support the assessment of pathogenicity of the variants. Metabolomics is promising in the diagnosis of *SDHx*-related disease. Future research should aim to further clarify the role of *SDHx* and *MAX* variants or other genes in the molecular pathogenesis of PitNETs, including pseudohypoxic and kinase signalling pathways along with elucidating epigenetic mechanisms to predict tumour behaviour.

## Introduction

Primary tumours of adenohypophyseal cells recently suggested to be redefined as pituitary neuroendocrine tumours (PitNETs) can rarely occur in association with paraganglioma (PGL) or phaeochromocytoma. These tumours may develop in patients with or without identifiable germline variants. The combination of PitNET and phaeochromocytoma/PGL (PPGL) is also uncommon but well-described in the setting of multiple endocrine neoplasia (MEN) type 1 whilst the association in MEN2 is probably coincidental. Succinate dehydrogenase (SDH) gene variants (collectively known as *SDHx*) can associate with PPGL (Baysal *et al.* 2000). The association of PitNET and PPGL in the setting of *SDHx* variant was established at the molecular level in 2012 (Xekouki *et al.* 2012) and has since been known as the 3P (pituitary, paraganglioma, phaeochromocytoma) association (3PA) syndrome (Xekouki *et al.* 2015). In some cases, no genetic alteration can be identified (Denes *et al.* 2015). In addition to PPGL and PitNETs, *SDHx* variants may also result in renal cell carcinoma and gastrointestinal stromal tumour (Carney & Stratakis 2002, Malinoc *et al.* 2012). The lifetime PPGL-related penetrance of *SDHA*, *SDHB* and *SDHC* genes is 1.7, 8.3 and 22.0%, respectively (Benn *et al.* 2018), while the penetrance of a paternally inherited *SDHD* pathogenic variant is 43.2% by age 60 years (Andrews *et al.* 2018). In decreasing order of frequency, germline mutations of *SDHx* genes have been found in PPGL, gastrointestinal stromal tumours, renal cell carcinoma and PitNETs, seemingly making PitNETs the least frequent of these *SDHx*-associated tumours (Evenepoel *et al.* 2015). Exceptional reports of *SDHx* variants in a pancreatic neuroendocrine tumour and lymphoid malignancy have been documented (Renella *et al.* 2014, Niemeijer *et al.* 2015). In unselected PitNET cohorts, the prevalence of *SDHx* variants is 0.3–1.8% (Gill *et al.* 2014, Xekouki *et al.* 2015, MacFarlane *et al.* 2020, Mougel *et al.* 2020).

Germline *MAX* variants have been implicated in PPGL and renal oncocytoma, and somatic variants have been identified in small cell carcinoma of the lung (Romero *et al.* 2014, Kurschner *et al.* 2017). Other tumours reported in association with *MAX* variants include endometrial carcinoma, ganglioneuromas, neuroblastoma, pancreatic cancer, lung adenocarcinoma and breast cancer (Walker *et al.* 2018, Seabrook *et al.* 2021). In one study, germline *MAX* variants accounted for approximately 1% of PPGLs in patients with a negative RET, VHL, SDHB, SDHC, SDHD and TMEM127 genetic screen, thus making it a very rare cause of PPGL (Burnichon *et al.* 2012). Data would tend to suggest that the presence of young onset bilateral PPGL or multifocal uniglandular phaeochromocytoma should raise the suspicion of a pathogenic *MAX* variant (Burnichon *et al.* 2012, Korpershoek *et al.* 2016, Seabrook *et al.* 2021). The 3PA syndrome has now also been described in patients with MYC-associated factor X gene (*MAX*) variants (Roszko *et al.* 2017, Daly *et al.* 2018, Mamedova *et al.* 2021). Two families with PPGL and multiple endocrine and non-endocrine tumours in the setting of *MAX* variants have raised the suggestion of naming this syndrome as multiple endocrine neoplasia type 5 (Seabrook *et al.* 2021).

This review summarises the inheritance and pathophysiology of *SDHx* and *MAX* variants, considers the clinical manifestations and discusses the evidence in reported cases of *SDHx* and *MAX-*associated PitNETs to date in order to provide an overview of the investigative strategy for these rare tumours.

## SDH: pathophysiology

**Figure 1:**
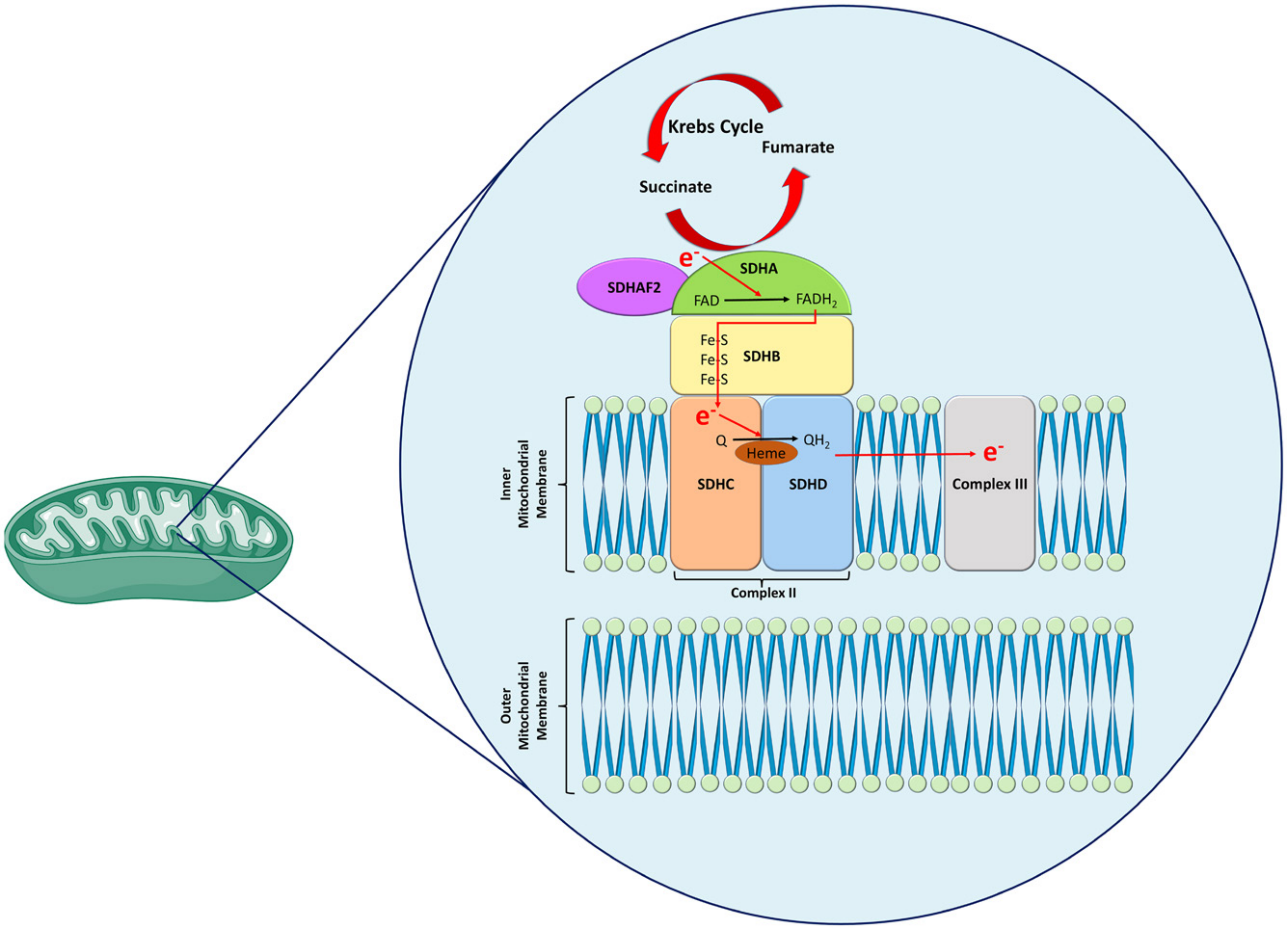
The SDH complex and its relationship to the mitochondrial membranes and mitochondrial cristae. Together the SDH subunits make up respiratory complex II. The hydrophobic SDHD and SDHC subunits anchor the complex within the inner mitochondrial membrane, while the hydrophilic SDHA and SDHB subunits catalyse the oxidation of succinate to fumarate as part of the tricarboxylic cycle. SDHAF2, also known as SDH5, is known to have roles in the flavination of SDHA and research into its roles and structure is ongoing (Sharma *et al.* 2020). Electrons generated by the tricarboxylic acid cycle (e-) reduce FAD to FADH_2_ in SDHA before proceeding through Fe-S clusters in SDHB. These electrons then reduce ubiquinone (Q) to ubiquinol (QH_2_) before being transported to the adjacent respiratory complex III. Mitochondrion image created with Biorender.com

The SDH complex is located on the inner mitochondrial membrane and consists of four subunits: A, B, C and D, each coded by one of the *SDHx* genes (Fig. 1). The SDH complex is accompanied by an associated assembly factor, SDHAF2, which facilitates flavination of SDHA (Fig. 1). The hydrophobic C and D subunits act to anchor the SDH complex, and the hydrophilic A and B subunits are the sites for enzymatic activity (Fig. 1). SDHA and SDHB catalyse the oxidation of succinate to fumarate in the tricarboxylic acid cycle (also known as Krebs cycle or citric acid cycle) and transfer electrons from carbon oxidation within the cycle to ubiquinone within the electron transport chain (Fig. 1) (Rutter *et al.* 2010). With its roles in both the tricarboxylic acid cycle and the electron transport chain, the SDH complex is a linchpin of aerobic respiration.

The malfunctioning of the SDH complex secondary to *SDHx* mutations results in accumulation of succinate. Accumulated succinate can then enter the cytosol via the inner mitochondrial membrane dicarboxylic acid translocator followed by the outer mitochondrial membrane voltage-dependent anion channel (Selak *et al.* 2005). This excess of succinate can disrupt prolyl hydroxylases within the cytosol resulting in the von Hippel–Lindau (VHL) protein dissociating from hypoxia-inducible factor (HIF) (Selak *et al.* 2005). The stabilisation and subsequent accumulation of HIF results in a state of pseudohypoxia, which may contribute to tumorigenesis via epigenetic modifications, such as disruption of RNA networks (Puissegur *et al.* 2011, Zhang *et al.* 2019). For example, a HIF-1α-dependent increase of miR-210 and subsequent mitochondrial dysfunction in A549 human lung adenocarcinoma cells has been demonstrated (Puissegur *et al.* 2011). Furthermore, SDHD is a miR-210 target and *SDHD* knockdown in A549 cells replicated miR-210-induced mitochondrial dysfunction and mitochondrial structural abnormalities (Puissegur *et al.* 2011). In another lung adenocarcinoma cell model (*EGFR*-mutated H1975 cells), miR-147b repressed SDHD activity, which is known to result in HIF accumulation (Zhang *et al.* 2019). Hypermethylation also appears to be an important epigenetic mechanism. In a cohort of 145 PPGL, only one hypermethylated tumour did not have an *SDHx* variant. Hypermethylation was higher in *SDHB*-mutated PPGL when compared to *SDHA*, *SDHC* and *SDHD* cases, which may explain the greater metastatic potential of* SDHB*-mutated tumours (Letouze *et al.* 2013). In this study, the authors hypothesised that succinate may limit demethylation by TET proteins and more recently it has been shown that inhibition of TET results in *SDHB*-related hypermethylation, which acts in concert with HIF-2α-induced pseudohypoxia to promote a mesenchymal phenotype in *Sdhb*^−/−^ cells *in vitro* and *in vivo* (Morin *et al.* 2020). Additionally, elevated HIF-1α levels have been shown in an *SDHD*-mutated somatotrophinoma and the cytoplasm of *Sdhb^+/−^* mouse pituitary cells (Xekouki *et al.* 2012, 2015).

The role of *SDHx* variants in pituitary tumorigenesis is supported by a double knockout animal model (Xekouki *et al.* 2015). *Sdhb*^+/−^ mice have hypercellular pituitary glands with increased number of prolactin and growth hormone-positive cells (Xekouki *et al.* 2015). Tumour cells in this model show large mitochondria with dysmorphic and/or absent mitochondrial cristae that are the site of SDH subunits (Fig. 1). It is hypothesised that pituitary hyperplasia could be one of the first steps in the development of *SDHx*-related PitNETs (Xekouki *et al.* 2015).

A transcriptomic analysis of 76 inherited and sporadic PPGLs identified 2 tumour clusters, one including *SDHB*, *SDHD* and *VHL*-mutated tumours (pseudohypoxic signalling cluster), and one comprising *RET* and *NF1*-mutated (kinase signalling cluster) tumours (Dahia *et al.* 2005). *MAX* falls within the kinase signalling cluster. A third cluster driven by Wnt signalling including *CSDE1* and *UBTF-MAML3* genes has also been recognised (Fishbein *et al.* 2017).

*SDHx* variants are well established in PitNETs while one highly proliferative macro somatotroph-lactotroph PitNET has been described in a 15-year-old with a germline* VHL* variant c.340G>C (p.Gly114Ser); the patient later developed a phaeochromocytoma (Tudorancea *et al.* 2012). It will be interesting to see if other genes identified in the pseudohypoxic cluster, such as *SUCLG2,* are also implicated in PitNET pathogenesis (Hadrava Vanova *et al.* 2022). Growth hormone excess in association with optic glioma and germline *NF1* variants has been reported, but a pathogenic role for *NF1* and *RET* germline variants is yet to be elucidated in PitNETs. Germline Wnt-signalling gene variants are yet to be described in PitNETs, although beta-catenin mutations are well established in the pathology of adamantinomatous craniopharyngioma.

The majority of *SDHx*-associated PitNETs reported to date have been tumours of the PIT1 lineage. This may be because PIT1 lineage PitNETs are simply more common, or alternatively, there may be a mechanistic explanation for this. For example, HIF-1 has many binding partners, one of these being the pituitary transcription factor *PITX1* (Mudie *et al.* 2014). PITX1 has been found to regulate HIF-dependent cellular survival in hypoxia and depletion of PITX1 in U2OS and HeLa cells resulted in increased apoptosis in hypoxic conditions (Mudie *et al.* 2014). Whether the elevated HIF levels arising from *SDHx* pathogenic variants may also inhibit apoptosis of PIT1-derived pituitary cells resulting in hyperplasia progressing to overt tumorigenesis is an interesting consideration, and a recent study has established a link between HIF-1α excess and protein kinase A, CREB and downstream excess growth hormone secretion via repression of *PRKAR2B* transcription (Lucia *et al.* 2020).

## *SDHx* variants

**Figure 2:**
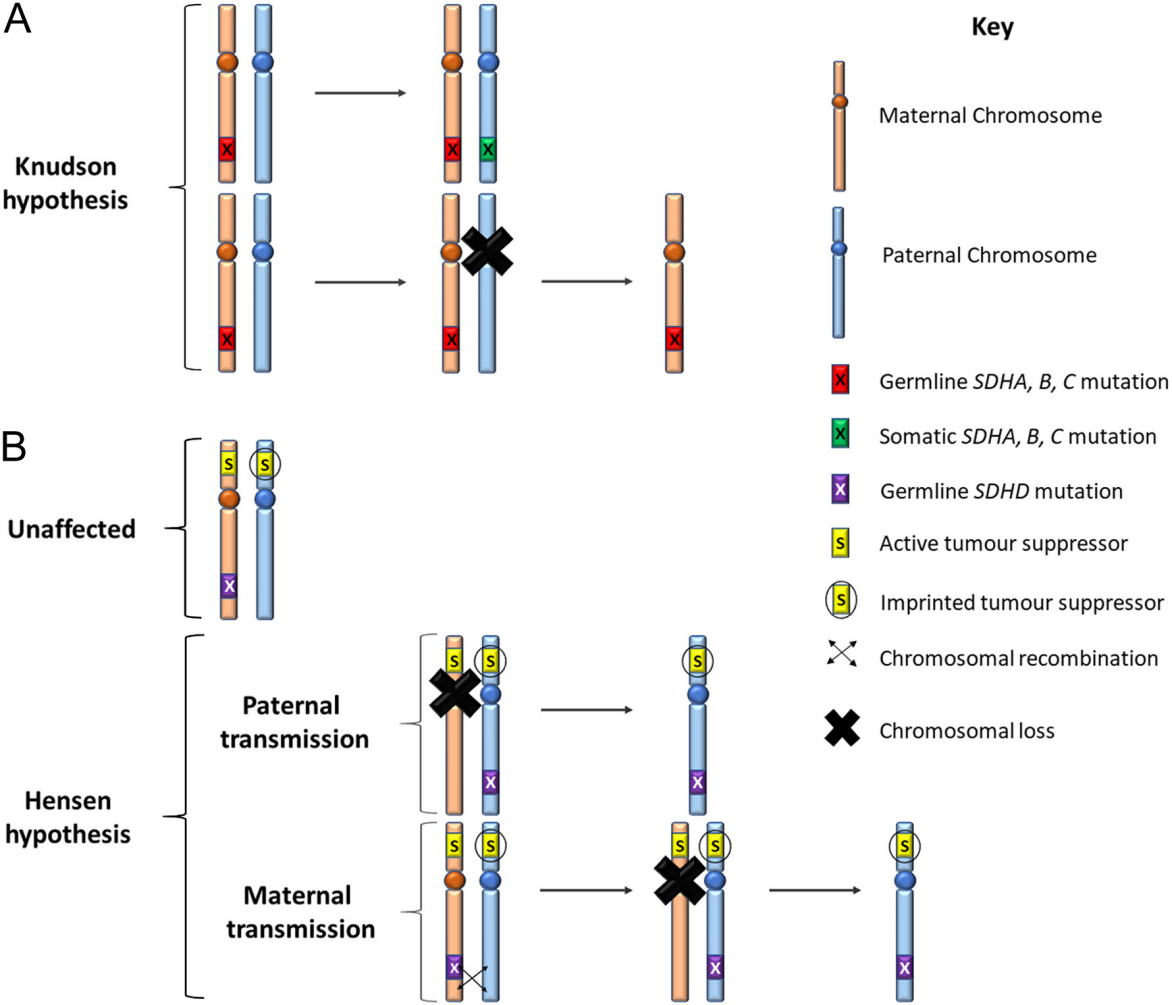
(A) The Knudson hypothesis. A germline mutation of SDHA, SDHB or SDHC is inherited and a second hit such as a somatic mutation or chromosomal loss is acquired resulting in loss of heterozygosity (LOH). (B) The Hensen hypothesis is applicable to SDHD (Hensen *et al.* 2004). SDHD is a maternally imprinted gene located on the long arm of chromosome 11. The presence of a paternally imprinted tumour suppressor gene (S) on the short arm is hypothesised. In the usual paternal transmission of SDHD, the mutated paternal SDHD together with the lack of expression of a tumour suppressor gene and loss of maternal chromosome 11 can result in tumorigenesis (B middle panel). If the SDHD mutation is on the maternal chromosome, usually no tumour is observed, as even if the paternal chromosome with the normal SDHD is lost, the lack of functional SDHD is counteracted by an expressed tumour suppressor gene from the short arm of the maternal chromosome 11 (B middle panel). In the rare maternal transmission of SDHD mutations, two steps are required. The first is suggested to be a chromosomal recombination resulting in transfer of the maternal SDHD mutation to the paternal allele harbouring the imprinted tumour suppressor gene. The second is loss of the maternal chromosome 11. The tumour suppressor gene S located on the short arm of chromosomal 11 is currently unknown. SDHAF2 also shows evidence of paternal imprinting (Kunst *et al.* 2011).

*SDHB*, *SDHA* and *SDHC* mutations are commonly inherited in an autosomal dominant fashion. Tumorigenesis in PPGL adheres to Knudson’s two-hit hypothesis (Fig. 2A). Patients with PPGL most commonly develop their disease from paternally transmitted mutations in *SDHD* and *SDHAF2*; however, a few cases of maternal transmission of *SDHD* mutations resulting in PPGL do exist (Kunst *et al.* 2011). Two different mechanisms have been suggested (Fig. 2B) (Hensen *et al.* 2004, Baysal *et al.* 2011). Proposed candidates for the unknown imprinted *SDHD* modifier gene shown in Fig. 2B include *CDKN1C, SLC22A18* and *H19* (Hoekstra *et al.* 2016, Björklund & Backman 2018).

**Figure 3:**
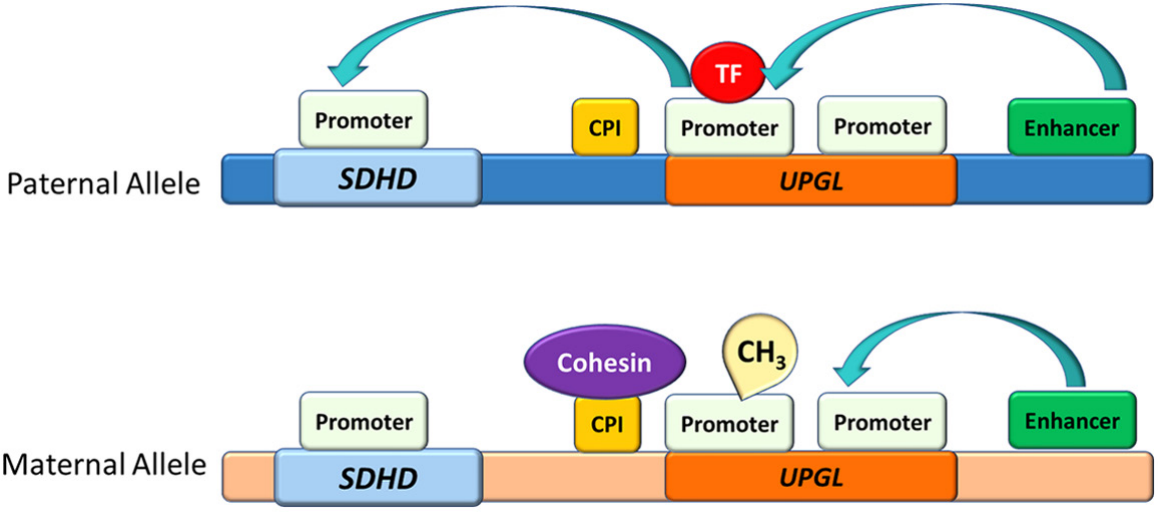
Long-range enhancer–promoter contacts in SDHD gene expression. On the paternal allele, an enhancer can influence an SDHD promoter and thereby increase SDHD transcription. This occurs via a UPGL promoter, which remains unmethylated due to the competitive binding of a transcription factor (TF) preventing cohesin from engaging with a CpG island (CPI). On the maternal allele, the UPGL promoter is methylated (CH3), preventing the TF binding, which enables cohesin to bind to the CpG island and block the enhancer–promoter activity on SDHD. Consequently, the enhancer binds to an alternative promoter and there is downregulation of SDHD transcription.

More recently, a further hypothesis for the parent-of-origin effects of *SDHD* expression suggested maternal imprinting at a promoter for a large intergenic ncRNA, designated the name *UPGL* (untranslated in paraganglioma locus) downstream of *SDHD* on chromosome 11 (Fig. 3) (Baysal *et al.* 2011). It is hypothesised that methylation of this locus controls long-range enhancer–promoter contacts, alteration of chromatin structures and subsequent downregulation of transcriptional activity of the *SDHD* gene (Baysal *et al.* 2011).

## *SDHB* variants in PitNETs

**Figure 4:**
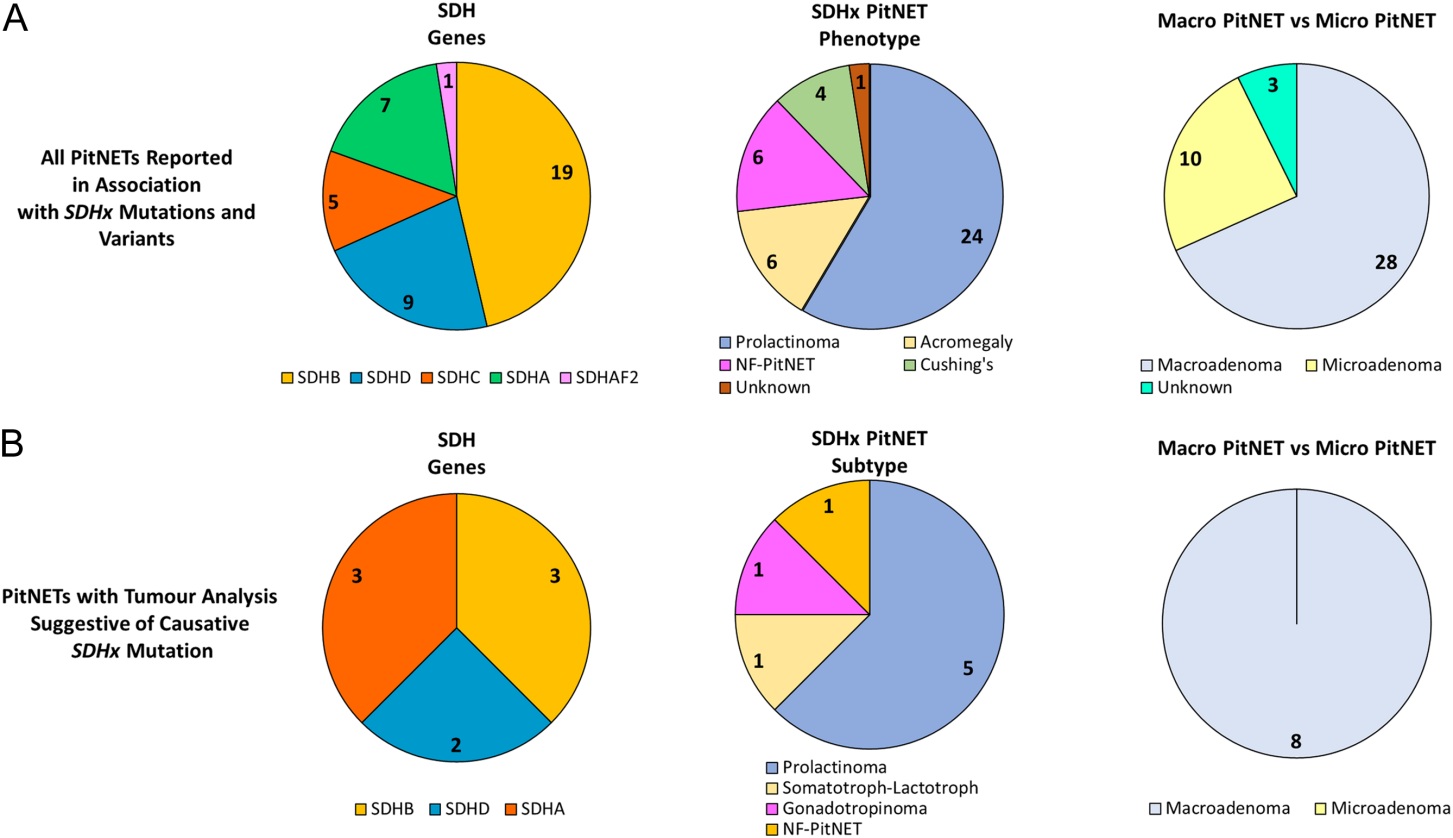
(A) All cases of PitNETs reported in association with SDHx variants are summarised. Prolactinomas account for a significant proportion (59%). It is notable that there have only been eight cases where evidence consistent with a causative role for SDHx variant reported in the literature and all are macro PitNETs (B). The average age at diagnosis in this sub-cohort is 44 years (range 31–60). One patient had a mixed somatotroph–lactotroph tumour with clinical acromegaly (Xekouki *et al.* 2012); 75% of this subgroup had prolactin-expressing tumours. It is also possible that prolactinomas will be under-represented in B as they are not routinely managed with surgery.

*SDHB* (OMIM*185470) is located on chromosome 1p36.13 and codes for the catalytic SDHB subunit of the SDH complex (Fig. 1). *SDHB* mutations manifest as familial PGL type 4. To date, there are 19 cases of *SDHB*-associated PitNETs reported. Five have had LOH analysis undertaken (three showed LOH). Evidence is inconclusive in the remainder of *SDHB*-related PitNETs analysed (LOH not present/not evaluated, heterogeneous/positive immunohistochemistry (IHC)). In 13 patients, no tissue analysis has been undertaken (Fig. 4 and Table 1). Cases with tumour analysis are summarised in this subsection.

**Table 1:** PitNETs reported in setting of *SDHx* variants.

Gene	*SDHx* variant	Sex	Age at diagnosis (years)	Phenotype	VarSome prediction	Reference
*SDHB*	c.761insC (p.254fs*255)	M	15	Unknown	Pathogenic	(Benn *et al.* 2006)
*SDHB*	c.18C>A (p.Ala6Ala)	F	43	Microprolactinoma	Benign	(Efstathiadou *et al.* 2014)
*SDHB*	c.423+1G>A (Splicesite)	F	60	Macroprolactinoma	Pathogenic	(Denes *et al.* 2015)
*SDHB*	c.770dupT (p.Asn258Glufs*17)	F	50	Micro NF PitNET	Pathogenic	(Denes *et al.* 2015)
*SDHB*	c.298T>C (p.Ser100Pro)	M	33	Macroprolactinoma	Pathogenic	(Denes *et al.* 2015)
*SDHB*	c.298T>C (p.Ser100Pro)	F	35	Macroprolactinoma	Pathogenic	(Denes *et al.* 2015)
*SDHB*	Deletion exon 6–8	F	31	Macroprolactinoma	Pathogenic	(Denes *et al.* 2015)
*SDHB*	c.587G>A (p.Cys196Tyr)	F	53	Gonadotroph carcinoma	Pathogenic	(Tufton *et al.* 2017)
*SDHB*	c.298T>C (p.Ser100Pro)	F	56	Macroprolactinoma	Pathogenic	(Maher *et al.* 2018)
*SDHB*	c.587-591DelC (Intronic)	F	74	Macro somatotrophinoma	Uncertain significance	(Saavedra *et al.* 2019)
*SDHB*	c.689G>A (p.Arg230His)	M	72	Somatotrophinoma	Pathogenic	(Xekouki *et al.* 2015)
*SDHB*	c.642+1G>A (p.Gln214His)	F	50	Microprolactinoma	Pathogenic	(Xekouki *et al.* 2015)
*SDHB*	c.487T>C (p.Ser163Pro)	F	14	Micro corticotrophinoma	Benign	(Xekouki *et al.* 2015)
*SDHB*	c.487T>C (p.Ser163Pro)	M	10	Micro corticotrophinoma	Benign	(Xekouki *et al.* 2015)
*SDHB*	Large deletion exon 1	F	38	Macroprolactinoma	Pathogenic	(Guerrero Pérez *et al.* 2016)
*SDHB*	c.5C>T (p.Ala2Val)	F	49	Microprolactinoma	Uncertain significance	(De Sousa *et al.* 2017)
*SDHB*	c.24C>T (p.Ser8Ser)	M	70	Prolactinoma	Benign	(De Sousa *et al.* 2017)
*SDHB*	Unknown	F	38	Macroprolactinoma	Unknown	(Gorospe *et al.* 2017)
*SDHB*	c.166-170delCCTA (p.Ala6Leu)	M	45	Macro NF PitNET*	Pathogenic	(Guerrero-Perez *et al.* 2019)
*SDHD*	c.298_301del (p.Thr100fs)	M	37	Macro somatotrophinoma	Pathogenic	(Xekouki *et al.* 2012)
*SDHD*	c.242C>T (p.Pro81Leu)	F	33	Macroprolactinoma	Pathogenic	(Varsavsky *et al.* 2013)
*SDHD*	c.274G>T (p.Asp92Tyr)	M	60	Macroprolactinoma	Pathogenic	(Papathomas *et al.* 2014)
*SDHD*	c.274G>T (p.Asp92Tyr)	F	56	Macro somatotrophinoma	Pathogenic	(Papathomas *et al.* 2014)
*SDHD*	c.149A>G (p.His50Arg)	F	16	Micro corticotrophinoma	Benign	(Xekouki *et al.* 2015)
*SDHD*	c.242C>T (p.Pro81Leu)	F	23	Macroprolactinoma	Pathogenic	(Xekouki *et al.* 2015)
*SDHD*	c.53C>T (p.Ala18Val)	M	12	Micro corticotrophinoma	Likely pathogenic	(Xekouki *et al.* 2015)
*SDHD*	c.315-?_480+?del	M	31	Macro NF PitNET	Pathogenic	(Lemelin *et al.* 2019)
*SDHC*	c.256–257insTTT (p.Phe85dup)	M	60	Macroprolactinoma	Uncertain significance	(Lopez-Jimenez *et al.* 2008)
*SDHC*	c.380A>G (p.His127Arg)	M	53	Macroprolactinoma	Likely pathogenic	(Denes *et al.* 2015) (Hussein *et al.* 2021)
*SDHC*	c.403G>C (p.Glu110Gln)	F	34	Microprolactinoma	Benign	(De Sousa *et al.* 2017)
*SDHC*	c.20+74A>G (Intronic)	M	41	Macroprolactinoma	Uncertain significance	(De Sousa *et al.* 2020)
*SDHC*	c.405+1G>T (Splicesite)	M	17	Macroprolactinoma	Pathogenic	(Mougel *et al.* 2020)
*SDHA*	c.1873C>T (p.His625Tyr)	M	30	Macro NF PitNET	Uncertain significance, likely pathogenic	(Dwight *et al.* 2013)
*SDHA*	c.725_736del (p.Ser243_Arg246del) and c.989_990insTA (p.Ala331ThrfsTer18)	M	62	Macro Silent Lactotroph PitNET	Likely pathogenic and pathogenic respectively	(Gill *et al.* 2014)
*SDHA*	c.969C>T (p.Gly323Gly)	M	53	NF PitNET	Benign	(Denes *et al.* 2015)
*SDHA*	c.91C>T (p.Arg31*)	F	27	Prolactinoma	Pathogenic	(Denes *et al.* 2015)**
*SDHA*	c.91C>T (p.Arg31*)	F	49	Macroprolactinoma	Pathogenic	(Niemeijer *et al.* 2015)
*SDHA*	c.757_758del (p.Val253Cys*67)	M	42	Macroprolactinoma	Pathogenic	(Mougel *et al.* 2020)
*SDHA*	c.1753C>T (p.Arg585Trp)	M	37	Macroprolactinoma	Uncertain significance	(Mougel *et al.* 2020)
*SDHAF2*	c.-52T>C (Intronic)	M	84	Macro somatotrophinoma	Uncertain significance	(Denes *et al.* 2015)

*This PitNET had focal positivity for prolactin and FSH. **The 27-year-old female with *SDHA* variant also had a concomitant *VHL* c.589G>A (p.Asp197Asn) variant.

A 33-year-old male with the *SDHB* c.298T>C (p.Ser100Pro) variant was reported to have a macroprolactinoma managed with dopamine agonist and surgery. LOH at the *SDHB* locus was confirmed in the tumour tissue, suggesting a pathogenic role of the *SDHB* variant (Denes *et al.* 2015). Furthermore, vacuoles were observed in neoplastic cells by microscopy. The patient’s mother carried the same variant and had been diagnosed with a macroprolactinoma aged 35 years. Her prolactinoma tissue also showed vacuolated cells (Denes *et al.* 2015).

A 31-year-old female with family history of PGL was diagnosed with macroprolactinoma requiring 2 surgeries, cabergoline and radiotherapy (Denes *et al.* 2015). She had a germline deletion of exon 6–8 of *SDHB*. The pituitary tissue showed loss of the whole gene on the other allele and negative SDHB IHC (Denes *et al.* 2015).

*SDHB-*associated pituitary carcinoma has been described in a 53-year-old patient bearing the c.587G>A (p.Cys196Tyr) variant (Tufton *et al.* 2017). The lesion was clinically non-functioning (NF). Tumour cells expressed the steroidogenic factor 1 (SF1) but lacked the expression of pituitary hormones. The patient also had a history of PGL. Vacuoles typical of *SDHB*-mutated PitNETs were identified and again LOH was confirmed in the pituitary carcinoma tissue (Tufton *et al.* 2017). After three cycles of temozolomide, the patient showed dramatic clinical improvement with stable MRI appearances. A slight reduction in the size of primary and metastatic lesions was noted after a total of ten cycles of chemotherapy.

In two further cases, the evidence for causation is considered inconclusive. One 56-year-old female patient bearing the *SDHB* c.298T>C (p.Ser100Pro) variant was diagnosed with macroprolactinoma (Maher *et al.* 2018). She had no syndromic disease. Her initial response to cabergoline was unsatisfactory. Surgical resection was undertaken. Histologically, the tumour cells showed considerable vacuolisation of the cytoplasm. The immunoreaction for SDHB showed normal expression suggesting that the *SDHB* variant might not have been causative and that a phenocopy was plausible. The most recent PitNET reported in association with an *SDHB* variant (c.587-591DelC frameshift) occurred in a 74-year-old female diagnosed with a macro somatotrophinoma on a background of metastatic PGL (Saavedra *et al.* 2019). Some neoplastic cells showed vacuoles. SDHA staining was retained whilst SDHB expression was reportedly heterogeneous from intensely positive immunostaining in some tumour cells to absent protein expression in others; no LOH was identified. The authors hypothesised this was a phenocopy, or alternatively, that partial loss of SDHB expression could have been pathogenic (Saavedra *et al.* 2019).

## *SDHD* variants in PitNETs

*SDHD* (OMIM*602690) is located on chromosome 11q23 and encodes the anchoring SDHD subunit (Fig. 1) (Baysal *et al.* 2000). Mutations in *SDHD* are responsible for familial PGL type 1. Maternal imprinting of this gene was presumed for some time due to the apparent exclusive paternal transmission of *SDHD* mutations. More recently, maternal transmission of *SDHx* mutations has been recognised to result in PGL (Figs 2B and 3) (Hensen *et al.* 2004, Yeap *et al.* 2011, Burnichon *et al.* 2017). The occurrence of a maternally inherited *SDHD* variant associated with PitNET has yet to be reported. There are currently eight cases of *SDHD*-related PitNETs in the literature, with an additional case described in this review. Of these nine patients, one had LOH, two had heterogeneous IHC and the majority (56%) did not have any analysis undertaken in tumour tissue (Table 1). The evidence for those cases subjected to a more in-depth analysis is discussed later.

The first *SDHD* variant-linked PitNET was reported in 2012 in a 37-year-old male diagnosed with somatotrophinoma and the c.298_301del (p.Thr100fs) variant (Xekouki *et al.* 2012). SDHD IHC showed reduced and patchy SDHD expression. LOH was identified. Two other patients were reported in 2014. A 60-year-old male with macroprolactinoma had the c.274G>T (p.Asp92Tyr) variant. Tumour cells lacked SDHB staining at IHC but expressed SDHA; preserved SDHA IHC being a recognised phenomenon in *SDHB*, *SDHC* and *SDHD* pathogenic variants (Oudijk *et al.* 2019). LOH was present. The evidence thus suggests a causative role of the *SDHD* variant (Papathomas *et al.* 2014). The second patient was a 56-year-old female with the same *SDHD* c.274G>T (p.Asp92Tyr) variant. She was diagnosed with macro somatotrophinoma. SDHA and SDHB expression was retained in tumour cells. No LOH was identified in the PitNET (Papathomas *et al.* 2014).

## *SDHC* variants in PitNETs

SDHC is one of the anchoring subunits of the SDH complex. The gene (OMIM*602413) is mapped on chromosome 1q23.3 (Fig. 1). *SDHC*-associated PitNETs are less frequently reported (*n* = 5) than those associated with *SDHB* and *SDHD* variants. To date, there has been no comprehensive report of PitNET secondary to a pathogenic *SDHC* variant (Fig. 4). The first PitNET associated with *SDHC* variant was reported in 2008. No IHC or LOH analysis was undertaken in tumour tissue (Table 1) (Lopez-Jimenez *et al.* 2008). Two cases were reported in 2017 (De Sousa *et al.* 2017). A 34-year-old female with a microprolactinoma and a 63-year-old female with pituitary gangliocytoma and primary hyperparathyroidism (De Sousa *et al.* 2017). Both cases carried an *SDHC* variant of unknown significance c.403G>C (p.Glu110Gln, VarSome predicted benign). However, no GH expression was identified in the gangliocytoma by De Sousa and colleagues and no somatotrophinoma was present in the tissue submitted for pathological assessment. In addition, the expression of growth hormone-releasing hormone was not evaluated in tumour tissue. The microprolactinoma expressed SDHB by IHC, reinforcing the prediction that this is a benign variant (De Sousa *et al.* 2017). The most recently described patient was a 17-year-old male with cystic macroprolactinoma and the pathogenic variant c.405+1G>T (splicesite) (Mougel *et al.* 2020). Tumour cells expressed SDHB. No cytoplasmic vacuoles were present and no LOH was proven suggesting this case might be a phenocopy (Mougel *et al.* 2020). Other cases of *SDHC*-associated PitNETs have been described but without supportive tissue analysis (Table 1).

## *SDHA* variants in PitNETs

*SDHA* (OMIM*600857) is located on chromosome 5p15.33. To our knowledge, seven cases of PitNET in setting of *SDHA* variants have been reported (Dwight *et al.* 2013, Gill *et al.* 2014, Denes *et al.* 2015, Niemeijer *et al.* 2015). Of these cases, two had LOH and three had no SDHA and SDHB expression in neoplastic cells.

The patient described by Gill and colleagues was a 62-year-old male with a 30 mm cystic, clinically NF-PitNET (Gill *et al.* 2014). Neoplastic cells were stained for prolactin and SDHA, whilst no staining for SDHB was present. No cytoplasmic vacuoles were described. Further analysis identified two inactivating somatic variants; a deletion on exon 6 (c.725_736del) and an insertion on exon 8 (c.989_990insTA) (Gill *et al.* 2014).

Another *SDHA* variant c.969C>T (p.Gly323Gly) variant (synonymous variant, predicted benign) was reported in a 53-year-old patient with an NF-PitNET and family history of NF-PitNET (father). The same patient had a history of nephroblastoma at the age of 1 year, 2 liposarcomas at 32 and 40 years, retroperitoneal PGL and renal oncocytoma both at the age of 50 years (Denes *et al.* 2015). The tissue from his PitNET did not show LOH or loss of SDHA and SDHB expression, suggesting that the *SDHA* variant was not causative. The variant c.969C>T was absent in his father’s NF-PitNET (Denes *et al.* 2015).

A male with *SDHA* c.1873C>T (p.His625Tyr) variant (VarSome uncertain significance, likely pathogenic) was diagnosed with NF-PitNET at the age 30 years (Dwight *et al.* 2013). SDHA and SDHB IHC showed no expression in the PitNET tissue. Paradoxically, the WT allele was retained; however, the authors suggested this might have been due to insufficient DNA to complete the analysis therefore missing the presence of an additional somatic second hit or alternatively failing to detect an epigenetic modification of the WT allele (Dwight *et al.* 2013). A 49-year-old female with *SDHA* c.91C>T (p.Arg31Ter) variant and macroprolactinoma was reported by Niemeijer and colleagues. Tumour tissue showed no SDHB and SDHA expression alongside LOH, suggesting the *SDHA* variant was contributory (Niemeijer *et al.* 2015).

The most recent case of *SDHA* variant was reported in a 37-year-old male with *SDHA* c.1753C>T (p.Arg585Trp) variant and macroprolactinoma. Surgery was undertaken due to poor compliance with medical therapy. Analysis of the tissue revealed SDHB staining, no vacuoles and no LOH, suggesting a phenocopy (Mougel *et al.* 2020).

## *SDHAF2* variants in PitNETs

The gene encoding the SDH assembly factor 2 (OMIM*613019) is mapped on chromosome 11q12.2. To our knowledge, no evidence supportive of a causative *SDHAF2* variant in PitNETs has been reported (Fig. 4).

## PitNET and PPGL in the setting of *SDHx* variant without tumour analysis

Many other reports have described PitNETs with PPGL associated with *SDHx* variant without tissue-based analysis to support a causative role for an *SDHx* variant. It is therefore possible that a considerable proportion of these cases could be phenocopies. A summary of *SDHx-*associated PitNET and PPGL including such cases is outlined in Table 1.

## MAX: pathophysiology

**Figure 5:**
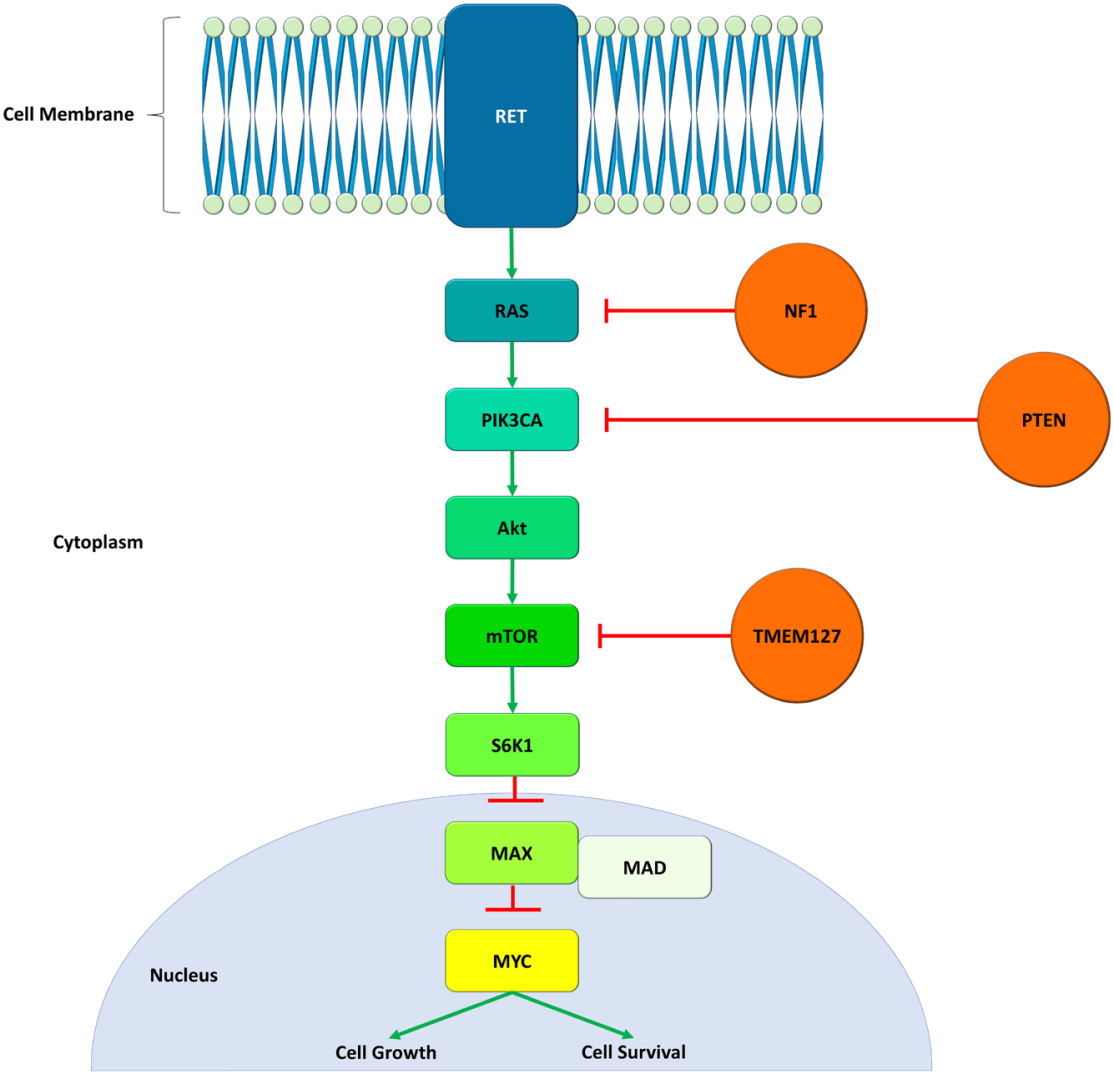
The role of MAX in tumorigenesis: MAX heterodimerises with MAD transcription factors and acts to repress the oncogenic MYC protein. MAX mutations impair heterodimerisation resulting in downstream unchecked MYC activity. Germline variants of RET, NF1, PTEN and TMEM127 have been implicated in PPGL, but not in PitNETs. As shown in the figure, the proteins they transcribe have roles in the MYC pathway.

*MAX* codes for the MAX protein, a component of the MYC signalling pathway. The protein forms heterodimers with C-MYC via basic-helix-loop-helix zipper (bHLHZ) domain interactions. These heterodimers can then bind to target DNA sequences or E-BOX sequences to regulate transcription of genes involved in cell proliferation and cell growth (Fig. 5). Like *SDHx*, epigenetics may play a role in *MAX*-associated tumorigenesis. Notably, the same microRNA (miR-210) implicated in *SDHx*-related disease has roles in MNT/MAX/MYC-mediated cellular proliferation (Walker *et al.* 2005, Zhang *et al.* 2009).

## *MAX* variants in PitNETs

*MAX* is located on chromosome 14q23.3 and appears to behave as a tumour suppressor gene with inactivating mutations resulting in a failure of dimerisation with MYC and unchecked downstream gene transcription. Germline and somatic *MAX* variants can result in familial and sporadic PPGL, respectively (Burnichon *et al.* 2012). *MAX* variants have been reported in the setting of uniparental disomy, with a tendency towards paternal transmission, like that seen in *SDHD* and *SDHAF2* (Comino-Méndez *et al.* 2011, Burnichon *et al.* 2012).

PitNETs have been reported in the setting of germline *MAX* variants. The possibility of MAX-associated syndromic disease being defined as multiple endocrine neoplasia type 5 has been mooted (Seabrook *et al.* 2021). Sporadic isolated or familial isolated pituitary adenoma in association with *MAX* variant has not yet been reported. One possible case of familial acromegaly with germline *MAX* variant (c.223C>T (p.Arg75Ter), VarSome pathogenic) has been documented (Mamedova *et al.* 2021), but the details on transmission are limited as the proband’s father was deceased. Based on old photographs showing acromegalic features, a history of receiving pituitary radiotherapy and sudden death (classical presentation of undiagnosed phaeochromocytoma), a familial syndromic disease with pituitary involvement in *MAX* germline variant seems possible (Mamedova *et al.* 2021).

Although microscopic features have not been reported for any *MAX*-associated PitNET, it appears they belong to the PIT1 lineage (prolactinomas and somatotrophinomas) (Roszko *et al.* 2017, Daly *et al.* 2018, Kobza *et al.* 2018, Seabrook *et al.* 2021). A report documented a 25-year-old presenting with hyperprolactinaemia responsive to cabergoline and a large PitNET. The patient re-presented at the age of 38 years with acromegaly. It is possible that the lesion was a mammosomatotroph or mixed somatotroph–lactotroph PitNET with growth hormone excess only becoming clinically evident later in life. The distinction between primary and secondary acromegaly has been a challenge in a kindred with a phaeochromocytoma expressing growth hormone-releasing hormone (Seabrook *et al.* 2021).

## Investigative strategy for tumours in the setting of *SDHx* and *MAX* variants

### Histopathological analysis

Cytoplasmic vacuoles and/or nuclear pseudo-inclusions and inclusions are a feature of *SDHx*-associated PitNETs (Denes *et al.* 2015, Tufton *et al.* 2017). Optically clear pseudo-inclusions are cytoplasmic invaginations into the nucleoplasm whilst inclusions result from the accumulations of proteins within the nucleus (Ip *et al.* 2010). The exact nature of nuclear inclusions can be difficult to establish, but they have been observed in the pituitaries of *Sdhb*^+/−^ mice (Xekouki *et al.* 2015). There is some evidence that *SDHx* variants can have structural and functional consequences on the mitochondrial assembly complex and the mitochondrial cristae (Gimenez-Roqueplo *et al.* 2001, Kim *et al.* 2015, Xekouki *et al.* 2015) and that fragmented mitochondria can be engulfed by cytoplasmic vacuoles before being extruded (Nakajima *et al.* 2008). Whether damage to mitochondria causes vacuoles and nuclear pseudo-inclusions/inclusions is yet to be proven (Tufton *et al.* 2017, MacFarlane *et al.* 2020). Neuropathologists should be aware of these morphological appearances and report tumours with prominent cytoplasm vacuolisation, raising the possibility of a germline *SDHx* variant as such a diagnosis has repercussions on genetic screening and familial counselling (Tufton *et al.* 2017). PitNET types and subtypes reported in association with *SDHx* variants include mainly prolactinomas, somatotrophinomas and clinically NF-PitNETs (Fig. 4). Five corticotroph PitNETs have been reported. Two in patients with a likely pathogenic variant and three in patients with likely non-pathogenic variants. Thyroid-stimulating hormone-secreting tumours are yet to be reported.

The introduction of lineage restricted pituitary transcription factors (PIT1, TPIT and SF1) and of GATA3 by immunohistochemistry will improve the identification of cell lineages of *SDHx*-associated NF-PitNET. There is little evidence to suggest that standard proliferative markers such as Ki-67 or mitotic count are increased in *SDHx*-mutated PitNETs. As mentioned, the light microscopic features of PitNETs in the setting of a germline *MAX* variants have never been documented. Clinically, *MAX*-associated PitNETs are similar to *SDHx* with a predominance of tumours causing hyperprolactinaemia and acromegaly.

### SDHA, SDHB and MAX IHC

Immunostains for SDHB and SDHA show positive granular cytoplasmic staining in non-*SDHx*-mutated cells (van Nederveen *et al.* 2009). Bi-allelic inactivation of any *SDHx* genes can result in degradation of SDHB. Absence or weak SDHB staining can therefore be supportive of *SDHx* variants being contributory to disease (Gill 2018). In one study, lack of SDHB expression at IHC demonstrated a sensitivity of 100% and specificity of 84% (van Nederveen *et al.* 2009). Studies have suggested that SDHB IHC can be positive in the setting of *SDHA* and *SDHD* variants. This finding is interesting and requires further investigation (Ugarte-Camara *et al.* 2019, Sato & Inomoto 2020, Snezhkina *et al.* 2020). False positive staining may account for this, but other possibilities include haploinsufficiency or a somatic mutation that may result in a dysfunctional SDH complex, which is still detectable by IHC (Ugarte-Camara *et al.* 2019). SDHB IHC is a cheap, reliable, readily available and quick test to screen tumours with vacuolar changes. However, a diagnostic algorithm suggested considering confirmatory functional tests (LOH or metabolomics) regardless of the SDHB IHC results, which can be employed as a screening step (MacFarlane *et al.* 2020).

The immunostain for MAX can also be used to assess its involvement in the pathogenesis. Expression in tumour cells theoretically refutes variant pathogenicity; however, in one of the studies, positive MAX IHC was seen in 3 out of 16 phaeochromocytomas with pathogenic *MAX* variant in the presence of LOH (Burnichon *et al.* 2012). This suggests, similarly to SDHB staining, a cautious and thorough approach to interpretation of MAX IHC should be considered.

### Loss of heterozygosity

LOH can support the tumorigenic role of a variant, but LOH is not confirmatory. In a small series of phaeochromocytomas, four of five *SDHB*-mutated and two of four *SDHD*-mutated cases demonstrated LOH, suggesting alternative genetic mechanisms (Weber *et al.* 2012). Methylation has been heavily implicated in *SDHx*-related disease as alternative mechanism causing silencing of the WT allele. Other possible mechanisms include haploinsufficiency or an additional variant in an alternative gene. Searching for LOH may hold more weight in tumours with *MAX* variants, with 16/18 tumours in one study demonstrating LOH and the 2 without LOH carrying *MAX* variants of unknown significance (Burnichon *et al.* 2012, Seabrook *et al.* 2021).

### Metabolomics

Metabolomics is a technique used to assess the biochemical functional status of cells/tissue samples via analysis of small molecule metabolites using NMR spectroscopy or mass spectroscopy and can be performed *ex vivo* or *in vivo*. The technique can be used in targeted and non-targeted approaches. *SDHx* mutations result in disruption of the SDH complex, leading to a break in Krebs cycle and accumulation of succinate. Therefore, succinate can be measured as a surrogate marker for defective SDH. Metabolomics with *in vivo* magnetic resonance spectroscopy has been utilised in assessing PitNET tissue of an *SDHB* variant carrier in one previous instance. Results did not show any accumulation of succinate (Casey *et al.* 2018). The following case description highlights the contribution of metabolomics in assessing the pathogenic function of *SDHx* variants.

#### Case description

**Figure 6:**
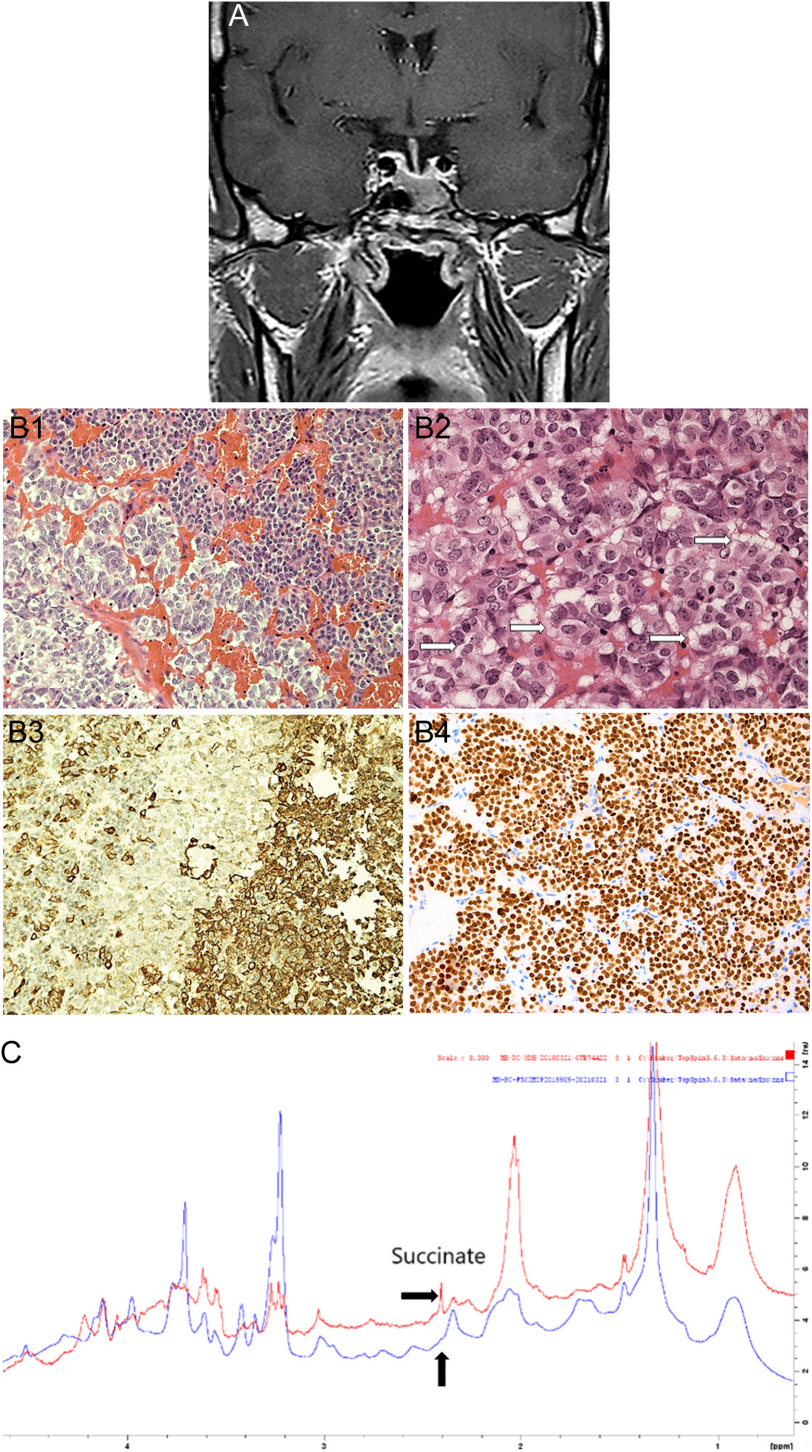
(A) Preoperative T1-weighted coronal MRI sequence displaying the extension in the left hemi-sinus. (B) Histopathologically, the tumour shows a biphasic pattern with a component of large cells with weakly eosinophilic cytoplasm (bottom left of the field) and a component with denser cellularity and cells with eosinophilic cytoplasm (top right of the field) (B1, HE – ×20); some tumour cells show vacuolated cytoplasm, indicated by white arrows (B2, HE – ×40); the immunostain for growth hormone highlights neoplastic somatotroph cells (B3, immunoperoxidase, ×20); Neoplastic cells show ubiquitous nuclear expression of the transcription factor PIT1 (B4, immunoperoxidase – ×20). (C) High-resolution NMR spectroscopy analysis shows the patient’s sample in blue in comparison to a control SDHx-mutated PGL sample in red. A succinate peak at 2.4 ppm is seen in the control PGL but is absent in the pituitary case, showing that the maternally inherited SDHD variant has not resulted in SDHD dysfunction.

A 32-year-old male with maternally inherited pathogenic *SDHD* c.242C>T (p.Pro81Leu) variant (Yeap *et al.* 2011, Xekouki *et al.* 2012, Denes *et al.* 2015) was diagnosed with acromegaly (insulin-like growth factor-1 105.3 nmol/L; age-adjusted reference range 11.6–32.2 nmol/L; nadir growth hormone 9.3 ng/mL on oral glucose tolerance testing) and concomitant hyperprolactinaemia (3182 mIU/L; reference range 63–245 mIU/L). He had secondary hypogonadism. The other tests of anterior pituitary function were normal. MRI revealed a large pituitary tumour invading the sphenoid sinus and eroding the clivus (Fig. 6A). No variants were found in the *AIP, MEN1* and* CDKN1B* genes. The patient’s father did not have *SDHx* variants. The patient underwent transsphenoidal surgery. Light microscopic features of the resected PitNET are shown in Fig. 6B.

Given the young age, tumour histotype and the presence of cytoplasmic vacuoles, the possibility that the *SDHD* variant resulted in the tumour was considered. In addition, the same *SDHD* exon 3 c.242C>T (p.Pro81Leu) missense variant resulting in maternally transmitted disease was also previously reported (Hensen *et al.* 2004, Denes *et al.* 2015, Xekouki *et al.* 2015). SDHB IHC showed normal expression and metabolomic profiling confirmed that the *SDHD* variant was not tumorigenic (Fig. 6B and C). This result has informed the future screening strategy for this patient and his family.

The performance of metabolomics appears far superior to SDHB IHC. The detection of succinate as an *SDHx*-related tumour screening test has shown remarkable sensitivity and specificity (Imperiale *et al.* 2015). The succinate:fumarate ratio also has excellent performance with sensitivity and specificity of 93% and 97%, respectively (Richter *et al.* 2014). In a recent assessment of IHC vs novel metabolomics and machine learning techniques, IHC resulted in a specificity of 86.7–93.8% in PPGL vs 99.2% of metabolomics (Wallace *et al.* 2020). The sensitivity of both techniques was comparable (85.2% for SDHB IHC and 88.1% for metabolomics) (Wallace *et al.* 2020).

Metabolomics has predominantly been applied to tumour tissue. Its application to liquid biopsy and the possibility of obtaining rapid results on urine or blood to detect accumulated metabolites circulated from *SDHx*-related tumours could change future clinical practice (Martins *et al.* 2019). However, further data and understanding of the peripheral metabolomics signatures of heterozygous carriers vs affected patients must be developed before the technique can be routinely implemented in the clinical setting.

### Management of *SDHx*-mutated PitNETs

Just over 50% of the PitNET tissue examined in the literature to date shows evidence of *SDHx* variants playing a role in tumour development. No comprehensive study performing whole exome or genome sequencing has been performed. Therefore, the causative role of an *SDHx* variant can only be confirmed in a small number of PitNETs. The evidence to suggest that *SDHx*-mutated PitNETs should be managed any differently to sporadic PitNETs is inconclusive. That said, it is evident that the incidence of PPGL in PitNET patients is significantly higher than expected (2 in 828 cases vs 0.33 expected) (Denes *et al.* 2015). Moreover, a mechanism has been established in an animal model suggesting that not all PitNET-PPGL cases are coincidental. Clinicians should be mindful of the potential for dual endocrine pathology and consider having a lower threshold to screen for PPGL in PitNET patients. In patients with PitNET in association with *SDHx* variant, it may be prudent to consider annual serum prolactin and insulin-like growth factor-1 levels during follow-up. In addition, screening MRIs should include imaging of the neck and may visualise some of the skull base so large PitNETs could be detected by standard* SDHx* variant radiological follow-up.

Sixty-seven percent of the reported *SDHx*-associated PitNETs and 100% of PitNETs with a causative *SDHx* variant were larger than 1 cm (macro) at diagnosis (Fig. 4), but tumour size and even invasion do not necessarily indicate aggressiveness. The pituitary carcinoma reported by Tufton and colleagues might indicate a potential aggressive behaviour of *SDHx*-mutated PitNETs (Tufton *et al.* 2017). Notably, both the carcinoma and metastases responded to the alkylating agent temozolomide. Resistance to first-generation somatostatin receptor ligand has been reported in one case (Xekouki *et al.* 2012). The majority of reported prolactinomas have responded well to dopamine agonists. In one case of macroprolactinoma and dopamine-secreting PGL in the setting of *SDHC* germline variant, the authors described the PGL responding to dopamine agonist therapy intended to treat the lactotroph PitNET. They highlighted the potential clinical pitfall of dopamine agonist therapy lowering 3-methoxytyramine levels and obscuring biochemical evidence of PGL metastases (Hussein *et al.* 2021).

The pseudohypoxia induced by accumulation of succinate is likely to cause changes in the tumour microenvironment. This may be a fruitful avenue for biomarkers and therapeutic targets in aggressive *SDHx-*mutated PitNETs. Metabolic profiling has documented increased levels of methionine, glutamine and myoinositol in *SDHx-*related PPGLs (Imperiale *et al.* 2015), indicating that targeting of metabolic pathways could have future therapeutic potential in these rare PitNETs.

### Management of *MAX*-associated PitNETs

There is not enough evidence on PitNETs in the setting of *MAX* germline variants to comment on management and on behaviour. *MAX*-associated lactotroph tumours seem to have a good biochemical response to dopamine agonists. The response of *MAX*-associated somatotroph tumours to somatostatin receptor ligands has been less convincing and multimodal therapies have been required (Roszko *et al.* 2017, Daly *et al.* 2018, Kobza *et al.* 2018, Seabrook *et al.* 2021).

## Conclusions

PitNETs caused by *SDHx* and *MAX* variants are rare. Several studies reported co-existing PPGL and PitNET with *SDHx* variant, but many of them did not perform tumour tissue analysis.

The immunoreactions for SDHB and MAX and LOH analysis are useful tools to support or refute the contribution of *SDHx* and *MAX* variants to disease, but these techniques have limitations. For this reason, metabolic profiling of SDH-associated disease is likely to have an important role in the future.

A vast amount remains to be learned about PitNET pathogenesis in the setting of *SDHx* and *MAX* variants and particularly on the role of pseudohypoxic and kinase signalling pathways in pituitary disease, which may reveal novel biomarkers and medical therapies. Pituitary tumours thought to be caused by *SDHx* and *MAX* variants are indeed rare. While the data does not firmly establish that the presence of these variants predicts future tumour behaviour, close follow-up of these patients would seem prudent.

## Declaration of interest

The authors declare that there is no conflict of interest that could be perceived as prejudicing the impartiality of this review.

## Funding

P B L is a clinical research fellow funded by Northern Ireland Health and Social Care Research and Development Division, Public Health Agency (EAT/5498/18).

## Consent for publication

Written informed consent has been obtained from the patient for publication of this report. We thank our patient for his consent and for his support.

## Author contribution statement

P B L, F R and M K wrote the manuscript. P B L, F R, E H, P W, R T C and S J H provided diagnostic support and clinical care of the patient. R T C and M B performed the metabolomics study. All authors reviewed and edited the manuscript.
